# Investigation of infectious droplet dispersion in a hospital examination room cooled by split-type air conditioner

**DOI:** 10.1007/s40201-024-00905-1

**Published:** 2024-05-29

**Authors:** Bahadır Erman Yüce, Onur Can Kalay, Fatih Karpat, Adem Alemdar, Şehime Gülsün Temel, Aslı Görek Dilektaşlı, Emel Bülbül Başkan, Cüneyt Özakın, Burhan Coşkun

**Affiliations:** 1 Department of Mechanical Engineering, Faculty of Engineering and Architecture, Bitlis Eren University, 13100 Bitlis, Türkiye; 2 Department of Air Conditioning and Refrigeration Technology, Bursa Uludag University, Yenişehir İbrahim Orhan Vocational School, 16900 Bursa, Türkiye; 3 Department of Mechanical Engineering, Faculty of Engineering, Bursa Uludag University, 16059 Bursa, Türkiye; 4 Department of Mechanical Engineering, Texas Tech University, Lubbock, TX 79409 USA; 5 Institute of Health Sciences, Department of Translational Medicine, Bursa Uludag University, 16059 Bursa, Türkiye; 6 Faculty of Medicine, Department of Medical Genetics, Bursa Uludag University, 16059 Bursa, Türkiye; 7 Faculty of Medicine, Department of Histology & Embryology, Bursa Uludag University, 16059 Bursa, Türkiye; 8 Faculty of Medicine, Department of Pulmonary Medicine, Bursa Uludag University, Bursa, Türkiye; 9 Faculty of Medicine, Department of Dermatology, Bursa Uludag University, Bursa, Türkiye; 10 Faculty of Medicine, Department of Infectious Diseases and Microbiology, Bursa Uludağ University, Bursa, Türkiye; 11 Faculty of Medicine, Department of Urology, Bursa Uludag University, Bursa, Türkiye

**Keywords:** Droplet Dispersion, Split-Type Air Conditioner, Computational Fluid Dynamics, Hospital Examination Room, Airborne Transmission, SARS-CoV-2

## Abstract

The novel coronavirus (SARS-CoV-2) outbreak has spread worldwide, and the World Health Organization (WHO) declared a global pandemic in March 2020. The transmission mechanism of SARS-CoV-2 in indoor environments has begun to be investigated in all aspects. In this regard, many numerical studies on social distancing and the protection of surgical masks against infection risk have neglected the evaporation of the particles. Meanwhile, a 1.83 m (6 feet) social distancing rule has been recommended to reduce the infection risk. However, it should be noted that most of the studies were conducted in static air conditions. Air movement in indoor environments is chaotic, and it is not easy to track all droplets in a ventilated room experimentally. Computational Fluid Dynamics (CFD) enables the tracking of all particles in a ventilated environment. This study numerically investigated the airborne transmission of infectious droplets in a hospital examination room cooled by a split-type air conditioner with the CFD method. Different inlet velocities (1, 2, 3 m/s) were considered and investigated separately. Besides, the hospital examination room is a model of one of the Bursa Uludag University Hospital examination rooms. The patient, doctor, and some furniture are modeled in the room. Particle diameters considered ranged from 2 to 2000 μm. The evaporation of the droplets is not neglected, and the predictions of particle tracks are shown. As a result, locations with a high infection risk were identified, and the findings that could guide the design/redesign of the hospital examination rooms were evaluated.

## Introduction

Respiratory diseases like Severe Acute Respiratory Syndrome (SARS), tuberculosis, Middle East Respiratory Syndrome (MERS), and the flu have led to significant economic losses worldwide and raised serious concerns due to the harm they inflict on individuals. [1]. The novel coronavirus (SARS-CoV-2), which recently emerged in Wuhan City, Hubei Province of China, and the risk of human-to-human infection is very high, poses a severe threat to public health [2]. Hence, the SARS-CoV-2 outbreak has spread worldwide and has been declared a global pandemic by the World Health Organization (WHO). Understanding the mechanism of droplet dispersion and evaluating them correctly, is important to minimize infection risk. In this way, it is possible to determine the most suitable ventilation designs and actual social distancing in hospitals and common areas where social interaction occurs. However, today even the transmission mechanism of the most common respiratory infection diseases is not fully understood.

Recent studies indicate that the infection of SARS-CoV-2 is transmitted by the inhalation of droplets emitted by speaking, coughing, sneezing (e.g., saliva and mucus) by other individuals or by contact with surfaces where the droplets adhere [3]. The distribution of droplets in the environment is characterized by droplet size and ambient airflow. The risk of infection transmission is directly related to the source patient and distance. Depending on the initial velocity, exhaled gas and particles can move a certain distance indoors, but the distance varies according to the ventilation airflow and particle diameter [4–6]. While particles with a diameter greater than 50 μm settle on the floor rapidly with the effect of gravity, particles smaller than 0.5 to 10 μm in diameter (droplet nuclei) remain suspended in the airflow for a long time, increasing the risk of infection [2]. Three transmission modes are discussed in the literature: self-inoculation, large droplet transmission, and airborne transmission. To reduce and control the spreading rate of SARS-CoV-2 via airborne transmission, the social distancing rule of approximately 1.83 m (6 feet) has been recommended. However, this distance was calculated based on static ambient airflow. Besides, the influence of relative humidity (RH) is not taken into account when determining traditional social distancing [7]. Many experimental studies in the literature examine the aerosol characteristics of the particles emitted as a result of coughing or sneezing [8–10]. Bourouiba et al. [11] visualized coughing and sneezing at different intensities by recording them with high-speed cameras in the appropriate lighting setup. Particle dispersion was investigated using the obtained image sequences. The findings have led to questioning the reliability of the social distancing recommended for indoor spaces. In reality, micro-droplets have low Stokes numbers [12] and can move much further than 1.83 m (6 feet) by the convection of the ambient airflow. Consequently, the role of environmental factors such as ventilation and RH in air transport of the SARS-CoV-2 and the infection risk need to be investigated.

In recent years, Computational Fluid Dynamics (CFD) has emerged as an effective and economical approach for an indoor microscopic examination compared to experimental methods. Chen and Zhao [13] investigated the dispersion characteristics of the droplets and employed the Euler–Lagrange-based method. The study observed that for particles between 0.1 to 200 μm in diameter, the initial exhaled velocity significantly affects particle distribution. In contrast, the effect of temperature and RH may be neglected. Li et al. [14] examined the distribution of particles emitted due to coughing by CFD and presented the Eulerian–Lagrangian-based method. Besides, important variables affecting particle dispersion were investigated and evaluated. Gupta et al. [15] examined particle transport in the aircraft cabin, an indoor environment with a high risk of infection. Particles were tracked using the Lagrangian model, and CFD analyzes were performed for different cases. Li et al. [16] investigated airborne contaminants in an aircraft cabin using numerical analysis. In the study, the numerical model was verified by using experimental data. Chen et al. [17] used a simple mouth-throat model and investigated the interaction of multicomponent droplets. Han et al. [18] examined the SARS outbreak case for an indoor study. The Euler–Lagrange method was employed, and the expiratory droplets were simulated. It was observed that the risk of infection was highly related to the movement behavior of the index patient. Liu et al. [19] studied the behavior of the expiratory particles to evaluate the infection risk and performed a water tank experiment. As a result, it has been determined that large-diameter particles are effective at a short distance. There is a risk of contamination in close contact with the infected person. Zhu et al. [20] investigated the transport characteristics of saliva droplets with different diameters, and the infection risk due to coughing was evaluated. Both experimental and analytical data were used in the study. It has been determined that droplets with a diameter of 50 to 200 μm were strongly affected by gravity. Vuorinen et al. [21] investigated the physics of aerosol and droplet dispersion. Besides, the 3D CFD simulations were conducted, and the effect of droplet size was evaluated for the supermarket case study. Qian and Li [22] examined the behavior of respiratory particles in a six-bed airborne infection isolation room. Both experimental and computational procedures investigated particle dispersion. The study suggested an improved ventilation design for the hospital isolation room. Sung et al. [23] carried out the tracer experiments for the 38 individuals infected in the MERS outbreak. As a result of the study carried out in a hospital in South Korea, it was indicated that precautions are essential in such cases and that airflow is a possible route of transmission. Bhattacharyya et al. [24] proposed a new method to sanitize the confined volume of the air for reducing the spread of SARS-CoV-2 in the hospital isolation room. Besides, the CFD analysis was performed to consider the factors affecting the aerosol sanitizer delivery system. Yuce [25] used the CFD method to examine the relation between elevator cabin size and droplet dispersion and analyzed transient simulation results to understand droplet pathways after sneezing activity in the cabin. Schumann et al. [26] conducted experimental and numerical studies in an operating room to compare the effectiveness of laminar air flow and turbulent mixing ventilation systems in reducing airborne germ and surgical smoke concentrations. Yuce et al. [27] investigated the optimization of ventilation parameters and room dimensions to minimize infectious pathogen dispersion.

The time people spend in indoor environments has increased due to rapid industrialization and urbanization. Besides, population growth and rising per capita income caused to use of air conditioners to be expanded. The split-type air conditioners are frequently preferred in Asian countries and Turkey due to their simplicity and effectiveness [28]. It is reported that Turkey’s split-type air conditioner sales in 2017 were approximately one million units [29]. On the other hand, split-type inverter air conditioners cover almost half of China’s domestic retail volume [30]. Respiratory infections can be transmitted to individuals through different transmission mechanisms in a critical environment, such as a hospital examination room. It is important to investigate the droplet dispersion in a hospital examination room cooled by a split-type air conditioner to understand the airborne transmission of SARS-CoV-2. In this way, precautions can be taken to reduce the spread of SARS-CoV-2 by identifying locations with high infection risk. Accordingly, the findings can guide redesigning old buildings and health institutions according to the possible infection risk or using appropriate ventilation system designs in new buildings to be built in the future.

In summary, visualizing particle dispersion is critical in terms of understanding human-to-human transmission of the virus and protecting people in the risk group (i.e., seniors, people with severe influenza, or diabetes). Besides, microscopic examination of the hospital examination rooms, where SARS-CoV-2 contamination risk is high, is of great importance considering the increasing number of deaths of healthcare workers and patients [31]. In this regard, it is also vital in terms of safety to determine the possible transmission routes of particles in a workplace where healthcare workers spend a long time.

This study numerically investigated the droplet dispersion emitted by the sneezing of a patient who was not wearing a face mask in a hospital examination room. The protective properties of masks against infection risk are a fact that has been demonstrated by many studies [4, 7, 32]. However, people may refuse to wear a mask or remove the mask reflexively when talking, coughing, or sneezing. For this reason, the patient was modeled without a mask to investigate the infection risk comprehensively. In addition, dynamic air movement is an important parameter that influences droplet dispersion. This study modeled a split-type air conditioner geometry to examine the effect of the air jet on the air conditioner. There are not enough numerical studies in the literature to fully understand infectious droplet dispersion. In this regard, the indoor environment should be addressed under different air conditioning and ventilation conditions. This study considered evaporation to improve the accuracy of numerical predictions, while many studies neglected the influence of evaporation [7]. Besides, the examination room geometry has its unique design, and it is divided into two-part with a wall except for a door space. This geometrical difference was also examined according to the droplet path lines. It aimed to contribute to knowledge in this field by examining droplet dispersion in a hospital examination room cooled by a split-type air conditioner. There is no similar study on split-type air conditioners in the literature within the authors’ knowledge.

## Materials and Methods

CFD provides the solution of nonlinear momentum, energy, and continuity equations by numerical methods. Thus, the flow properties in a certain domain can be investigated at each point of the volume. It is not easy to test turbulent flow, heat transfer, and droplet dispersion cases that strongly depend on flow patterns with experiments, but it is possible and cheaper with CFD. Besides, the CFD method enables tracking all particles in a certain volume.

RH, particle size, settling velocity, airflow, and types of organisms determine the distance traveled and the length of time particles remain suspended in the air [33]. The particles’ motion can be predicted to be chaotic due to the relationship between the flow physics of air and the motion of the particles. This study investigated the distribution of particles spread by the sneezing of a patient not wearing a mask in the examination room with the CFD method. A commercial software, ANSYS Fluent 20.0 (ANSYS Inc., Canonsburg, USA), was used to perform simulations.

The flow conditions, such as transient, turbulent, and compressible, were considered in the simulations. The ideal gas approach was used to calculate the natural convection heat transfer mechanism. The turbulence model in the simulations was considered the RNG k-ε turbulence model, and for the wall function approach, and the scalable wall function was selected for the wall function approach. This turbulence model and wall function selection were investigated in previous studies, and the turbulence model and wall function have a good performance when compared with experimental results in ventilation studies [34]. As a result, the computational cost was decreased, and a stable solution with smaller element numbers was obtained.

### Model

**Fig. 1:**
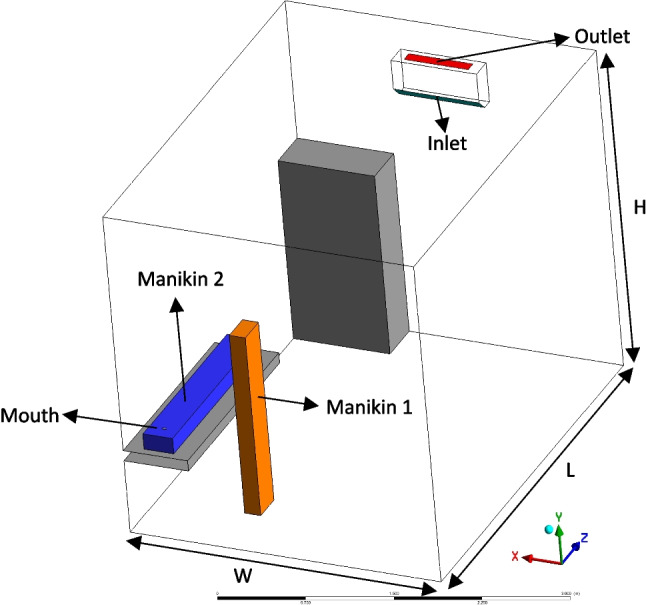
3D model of the Bursa Uludag University Hospital examination room

The dimensions of one of the standard-sized examination rooms in the Bursa Uludag University Hospital were used as geometry. The 3D model of the room is presented in Fig. 1. The furniture, stretcher, doctor, and patient are modeled in the room. All geometries are modeled as cubical to reduce computational cost. Besides, the split-type air conditioner is modeled as the air conditioning unit. The split-type air conditioner is a widely used air-conditioning system in Turkey and is also frequently used in hospital examination rooms. The air conditioner is located on the mid-plane (Z) of the room according to the XY plane. The room height (H) is 3.1 m, room width (W) is 2.9 m, and room length (L) is 3.795 m. The dimension of both manikins is 1.75 × 0.275x0.15 m. The air conditioner is 2.42 m high from the floor and positioned in the middle of the wall. The air conditioner dimensions are 0.8 × 0.38 × 0.2 m, and the blowing angle is modeled to be 45°.

In Fig. 1, manikin 1 represents the doctor, and manikin 2 represents an infected patient in the supine position on the examination table. For representing the mouth, a surface on the patient was modeled to simulate sneezing. Before the numerical solution process, the geometry is divided into finite volumes. Three element numbers (468,392, 656,975, 1,083,205) were used to obtain the grid-independent solution. As a result, the mesh domain with 656,975 hexahedral elements was preferred due to the solution stability. The grid independence analysis yielded more robust solutions, indicating that velocity, temperature, and droplet distribution results remain stable despite the increase in the number of elements.

The split-type air conditioner was assumed to work according to summer conditions; therefore, the environment's initial temperature was 28 °C. The blowing temperature of the air conditioner is 22 °C, and the blowing velocity is investigated with three different values 1, 2, and 3 m/s, and inlet velocity is vertical to the blowing surface. A cabinet and a stretcher are modeled in the examination room. Besides, all walls are at the initial air temperature of 28 °C. The manikin surface temperature was considered as 36.85 °C. The boundary conditions were determined according to Yamankaradeniz et al. [35].

At first, all simulations were performed in steady-state conditions without particle injection, and then transient boundary simulations were performed. The iterations were finalized in the 20 s because of hardware limitations. 0.002 s was applied as the time step size. 300 iterations were set for each time step. Except for the energy equation, the convergence criteria of all equations were set at 10^–6^, and 10^–9^ was applied for the energy equation.

The transport of virus-containing droplets expelled during sneezing was predicted using a multiphase model based on Euler–Lagrange equations. When sneezing, coughing, breathing heavily, or speaking rapidly, water droplets containing particles are expelled. The size of these droplets varies within a certain range, and these particles are not homogeneous in size [7, 33, 36]. The particle diameter range was set from 2 to 2000 μm [7]. and the distribution of droplets of different sizes was defined by a Rosin–Rammler distribution [37–39]. The droplet breakup model employed in this study is based on the Taylor Analogy Breakup (TAB) model, and unsteady particle tracking is used for the simulations to predict droplet trajectories [31]. The calculation of droplet trajectories involved solving the translation equation of the discrete phase while ignoring droplet rotation [7, 40]:

1 $$\frac{d}{dt}\left({m}_{d}{u}_{d,i}\right)={F}_{i}^{D}+{F}_{i}^{L}+{F}_{i}^{BM}+{F}_{i}^{G}$$

where ${F}_{i}^{L}$, ${F}_{i}^{D}$, ${F}_{i}^{BM}$, and ${F}_{i}^{G}$ are the lift force, drag force, Brownian motion-induced force, and gravity, respectively. In addition, ${F}_{i}^{D}$ can be calculated by the equation presented below:

2 $${F}_{i}^{D}=\frac{\frac{1}{8}\pi \rho {d}_{d}^{2}{C}_{D}\left(\overrightarrow{u}-{\overrightarrow{u}}_{d}\right)\left|\overrightarrow{u}-{\overrightarrow{u}}_{d}\right|}{{C}_{c}}$$

where ${C}_{c}$ is the Cunningham correction factor [7] and ${d}_{d}$ is the droplet diameter. In Eq. 2, ${C}_{D}$ is defined:

3 $${C}_{D}={a}_{1}+\frac{{a}_{2}}{{{\text{Re}}}_{d}}+\frac{{a}_{3}}{{{\text{Re}}}_{d}^{2}}$$

The constants ${a}_{1},{a}_{2}$ and ${a}_{3}$ are defined based on the Reynolds number of the droplet.

The evaporation and condensation between ambient water liquid in cough droplets and water vapors are considered by solving the mass and energy balance for each droplet [7, 41]:

4 $$\frac{d{m}_{d}}{dt}=-\sum_{e=1}^{k} {\int }_{\text{surf }} {n}_{e}dA\approx -\sum_{e=1}^{k} \left({\overline{n} }_{e}\cdot A\right)$$

In equation x, $k=1$ for water and ${\overline{n} }_{e}$ means average mass flux of evaporable component $e$ on the surface, which can be calculated as below [17]:

5 $${\overline{n} }_{e}=\frac{{\rho }_{g}{\text{Sh}}{\tilde{D}}_{e}{C}_{m}}{{d}_{d}}{\text{ln}}\frac{1-{Y}_{e,\infty }}{1-{Y}_{e,\text{ surf}}}$$

In the equation above ${\rho }_{g}$ represents the density of the ambient air, and $Sh$ is the Sherwood number [42] and can be calculated as below:

6 $$Sh=\sqrt[3]{1+{{\text{Re}}}_{d}\cdot Sc}\cdot {\text{max}}\left[1,{{\text{Re}}}_{d}^{0.077}\right]$$

In Eq. 6, $Sc=\frac{\mu }{\rho {D}_{e}}$ represents the Schmidt number, and ${D}_{e}$ means mass diffusivity of component $e$. In Eq. 5, ${Y}_{e,\infty }$ and ${Y}_{e,\text{ surf}}$ are the mass fractions of evaporable component $e$ in the gas phase far from the droplet and on the droplet surface respectively. ${C}_{{\text{m}}}$ represents the Fuchs-Knudsen number correction [43]:

7 $${C}_{m}=\frac{1+Kn}{1+\left(\frac{4}{3{\alpha }_{m}}+0.377\right)Kn+\frac{4}{3{a}_{m}}K{n}^{2}}$$

In Eq. 7, $Kn$ represents the Knudsen number ($Kn=2\lambda /{d}_{d}$). ${\alpha }_{m}$ is the mass thermal accommodation coefficient and $\lambda$ is the ratio between the the mean thermal velocity of the condensing water vapor and diffusion coefficient of water vapor. ${Y}_{e,\text{ surf}}$ can be calculated using the modified Raoult's law [17]

8 $${Y}_{e,\text{ surf }}={\gamma }_{e}{x}_{e}{K}_{e}\frac{{P}_{ve,sat}\left({T}_{d}\right)}{\rho {R}_{e}{T}_{d}}$$

where, ${x}_{e}$ is the mole fraction of $e$ in the droplet, ${\gamma }_{e}$ is the activity coefficient of component$e$, ${T}_{d}$ is the droplet temperature, ${R}_{e}$ is gas constant, ${P}_{ve,sat}\left({T}_{d}\right)$ is the saturation pressure of component $e$ at temperature${T}_{d}$, and ${K}_{e}$ is the correction factor for the Kelvin effect [12]. In addition,${K}_{e}$, mentioned in Eq. 8, can be calculated with equation below:

9 $${K}_{e}={\text{exp}}\left(\frac{4\sigma {M}_{e}}{R{\rho }_{d}{d}_{d}{T}_{d}}\right)$$

where ${\rho }_{d}$ is the droplet density, ${M}_{e}$ is the molar mass of component $e,R$ is the universal gas constant, and $\sigma$ is the surface tension of the droplet.

The equation for the energy balance of the droplet is presented as follows:

10 $$\sum_{i=1}^{m} {m}_{d,i}{c}_{d,i}\cdot\Delta T=\pi {d}_{d}{\lambda }_{g}Nu\left({T}_{a}-{T}_{d}\right)-\sum_{e=1}^{k} {\iint }_{d} {n}_{e}{L}_{e}dA$$

$Nu$ represents the Nusselt number and is defined below:

11 $$Nu={\left(1+{{\text{Re}}}_{d}{\text{Pr}}\right)}^{1/3}{\text{max}}\left[1,{{\text{Re}}}_{d}^{0.077}\right]$$

In Eq. 11, ${\text{Pr}}$ is the Prandtl number and ${{\text{Re}}}_{d}$ means Reynolds number of the droplet.

The sneezing velocity of 4.5 m/s was adopted from the literature review, and the drag force was calculated using the spherical drag law [44]. The droplets were initially set to a temperature of 36.5 °C, and a summary of the numerical simulation parameters is provided in Table 1.

**Table 1:** A summary of the boundary conditions

Parameter	Unit	Value
Environment temperature	°C	28
Air conditioner blowing temperature	°C	22
Air conditioner blowing velocity	m/s	2
Manikin temperature	°C	36.85
Diameter of the droplets	μm	2 – 2000
Sneezing velocity	m/s	4.5
Initial droplet temperature	°C	36.5

## Results

This study simulated the particle dispersion in the transient airflow emitted by the sneezing of a patient in the hospital examination room ventilated by a split-type air conditioner with different inlet velocities.

CFD is frequently used in ventilation and aerosol studies in the literature [13–15]. In this regard, many studies are examining ventilation and droplet distribution together. The number of studies conducted to understand the distribution of SARS-CoV-2 in indoor environments during the global pandemic has increased and become more significant.

Mechanical ventilation is not common enough in most countries' workplaces and public institutions. For this reason, numerical studies on droplet distribution will both help to understand the pattern of the spread of infectious droplets and raise public awareness of the importance of mechanical ventilation.

**Fig. 2:**
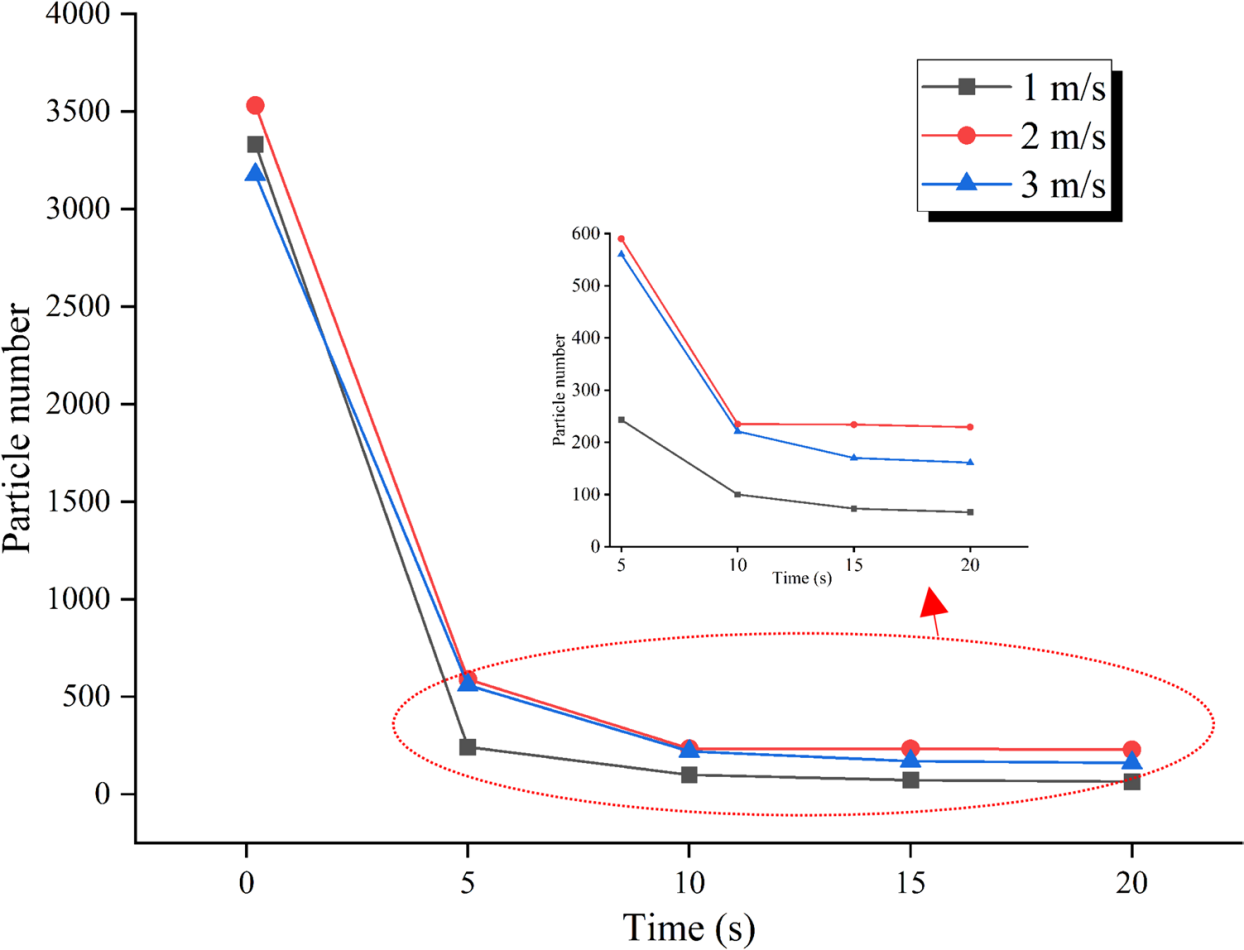
Time-dependent variation of airborne droplet number at different inlet velocities

**Fig. 3:**
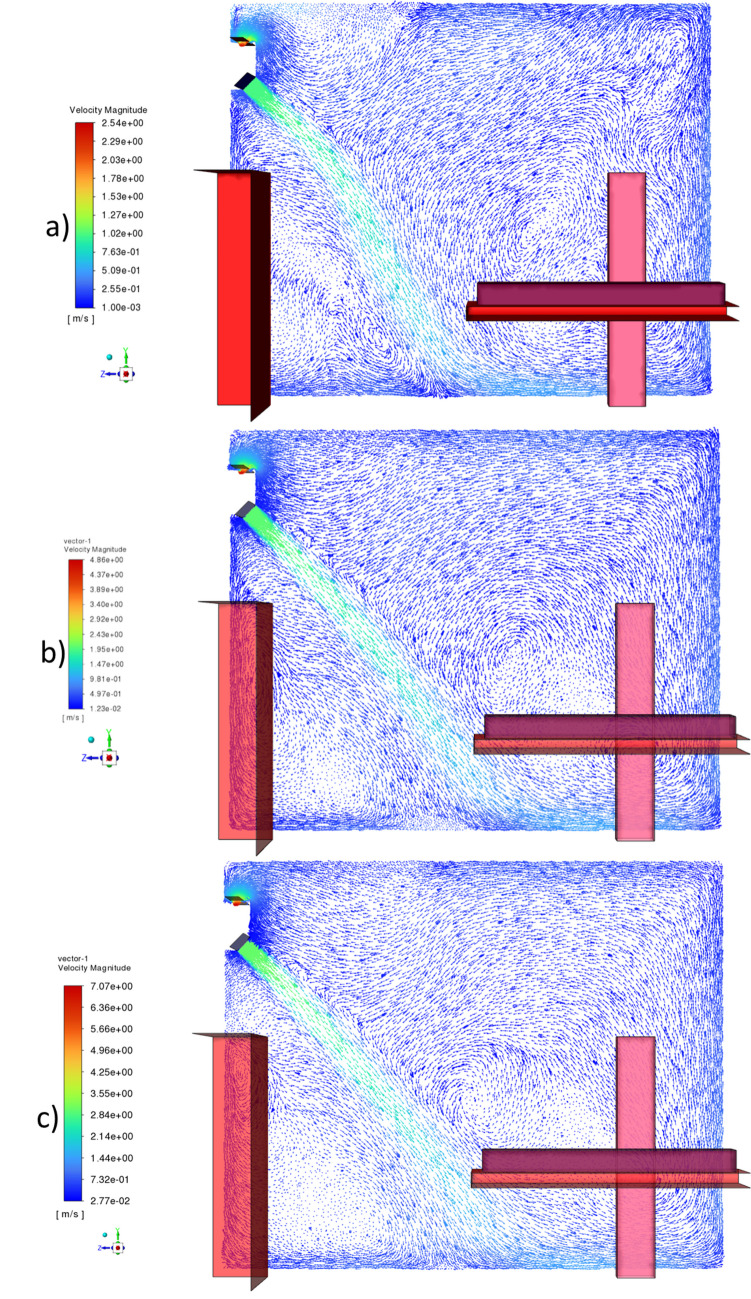
Vectoral velocity distribution for different inlet velocities: a) 1 m/s, b) 2 m/s, c) 3 m/s

The decrease of droplet number according to time steps at different inlet velocity cases is shown in Fig. 2. It is seen that at the low blowing speed of 1 m/s, the number of droplets in the room is at the lowest level in all time steps. The low blowing speed caused the air jet to be more unstable and the vortexes in the room to be less effective, as can be seen in Fig. 3. For this reason, the droplets are less affected by the air movement and are trapped on the surfaces with the effect of gravity. When the blowing speed was increased to 2 m/s, the number of droplets in the air increased significantly compared to 1 m/s. The reason for this result is that the airflow in the room, which is seen in Fig. 3, becomes stronger due to the increased blowing velocity. When the inlet velocity is increased to 3 m/s, which is the highest value examined, it is observed that the droplet number shows a similar behavior with 2 m/s until the 10th second, but the drop in the droplet number decreases after the 10th second. Due to the strong air flow seen in Fig. 3, the droplets were pushed to the surfaces more and trapped on these surfaces. For this reason, the total number of droplets in the air decreased compared to 2 m/s in 3 m/s blowing air.

**Fig. 4:**
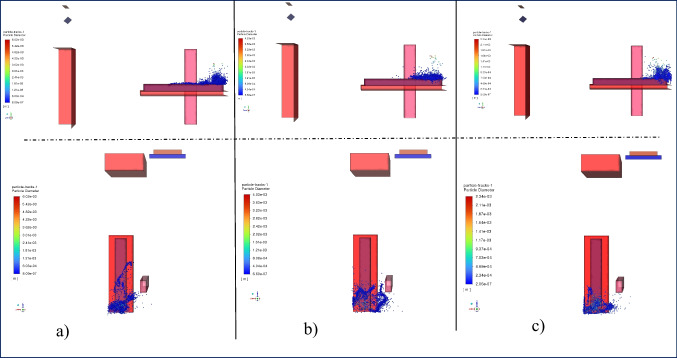
Particle distribution at t = 0.2 s on side and top view at a) 1 m/s, b) 2 m/s, c) 3 m/s inlet velocity

In Fig. 4, droplet dispersion at t = 0.2 s is shown according to different inlet velocities. Droplet injection was done equally for all conditions for 0.1 s, and up to 0.2 s, some of these droplets evaporated, adhered to the surface, or coalesced. As a result of blowing the inlet air at 1 m/s in 0.2 s, it is seen that the droplets spread over a wider area compared to other blowing velocities cases. As the blowing rate increased, the droplets were pushed against the wall behind the patient by airflow, forming a cluster around the patient's head. At an inlet velocity of 1 m/s, the droplets were distributed in the + z direction.

**Fig. 5:**
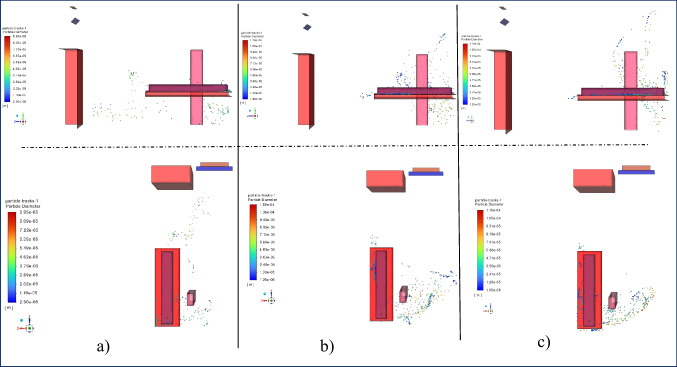
Particle distribution at t = 5 s on side and top view at a) 1 m/s, b) 2 m/s, c) 3 m/s inlet velocity

The droplet dispersion for t = 5 s is shown in Fig. 5. At this time step, it is seen that the droplets spread over a wider area at all blowing velocities compared to t = 0.2 s. The distribution in the + z direction at the 1 m/s inlet velocity seen in the previous case continued, spreading over a wider area. In this case, the spreading occurred mostly just below the jet formed by the inlet air. It is seen that droplets with a large diameter move toward the floor, while droplets with a small diameter move with the airflow. In the case of 2 m/s blowing air, there is a slight distribution in the + z direction compared to the previous time step, but the droplets were mostly clustered in the blowing direction (-z) of the air conditioner. Unlike the previous situation, it also created a distribution in the + y direction depending on the air movement of the small-diameter droplets. A similar situation is observed more clearly in the case of 3 m/s inlet air. At an inlet velocity of 3 m/s, droplets in the -z direction are higher due to the more effective air movement.

**Fig. 6:**
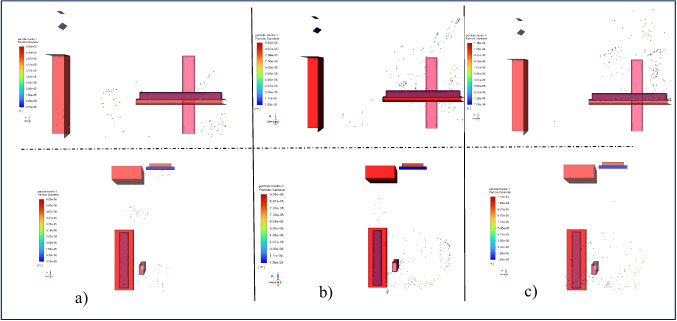
Particle distribution at t = 10 s on the side and top view at a) 1 m/s, b) 2 m/s, c) 3 m/s inlet velocity

Figure 6 shows the droplet spread at t = 10 s. At this time step, it is apparent that all droplets have spread over a larger volume. It was observed that the droplets continued to spread in the + z direction at an inlet velocity of 1 m/s, while their spread in the x and + y directions remained limited. The spread was observed to increase as the time steps advanced with a blowing speed of 2 m/s. Due to the smaller diameter of the remaining droplets, they became more sensitive to airflow, and most of them were positioned above the stretcher level. The spread increased in regions outside the air jet axis. At a blowing speed of 3 m/s, it was noted that most of the droplets had been positioned above the stretcher level. However, there was a decrease in droplet number density, but no significant changes were observed in the x and z axes in the droplet distribution.

**Fig. 7:**
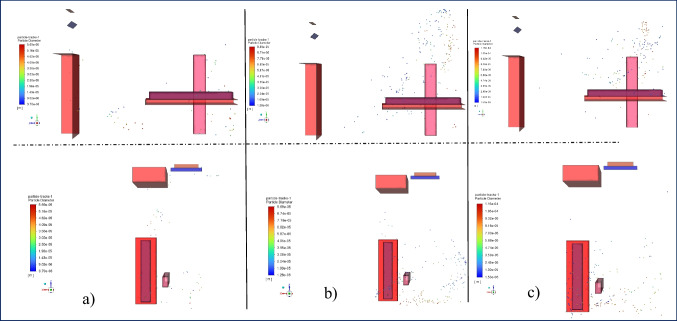
Particle distribution at t = 15 s on the side and top view at a) 1 m/s, b) 2 m/s, c) 3 m/s inlet velocity

**Fig. 8:**
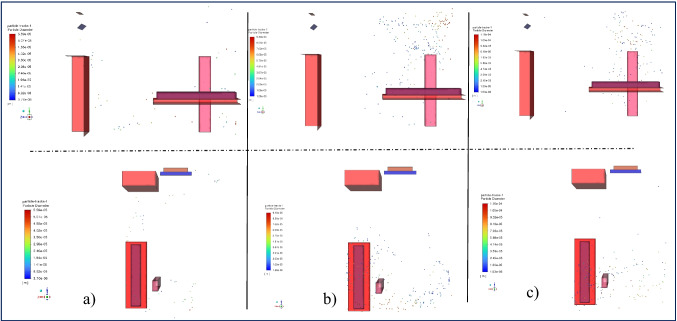
Particle distribution at t = 20 s on the side and top view at a) 1 m/s, b) 2 m/s, c) 3 m/s inlet velocity

The spreading of droplets at the 15th and 20th seconds is illustrated in Fig. 7 and Fig. 8, respectively. The results are pretty close in these time steps. As shown in Fig. 2, except for the 2 m/s blowing air, a decrease in the number of droplets continues at these time steps, and in the case of 2 m/s, this decrease is slower. It is observed that there is no significant change in droplet spreading at the lowest blowing velocity of 1 m/s. The distances between the droplets have increased, and thus, the space they occupy has also increased. The droplets move in the y + direction due to the rotational motion of the air created by the position of the blowing and suction vents of the air conditioner at a blowing velocity of 2 m/s. Therefore, the motion continues in the y + direction. However, the space between the droplets in the -x direction has increased, resulting in increased spreading. A similar situation was observed for the 3 m/s inlet velocity.

## Conclusions

Airflow in turbulent flow is chaotic; consequently, particle dispersion prediction is impossible without numerical simulations. Every closed space and scenario has its conditions and should be investigated separately. In this present study, droplet dispersion from the sneezing of a patient in a hospital examination room is investigated numerically using the CFD method under different conditions. The room is ventilated by a split-type air conditioner, and the air conditioner affects the droplet dispersion and infection risk with the air jet. The following conclusions can be drawn.

1. Droplets emitted by the sneezing of a patient in the supine position on the examination table and not wearing a mask were evaporated, trapped, or escaped from the ventilation outlet. A 98.02%, 93.51%, and 94.83% decrease occurred in droplet numbers in cases with different inlet velocity values as 1 m/s, 2 m/s, and 3 m/s, respectively.
2. Changing the inlet velocity directly affects the droplet concentration in the air. Turbulence is a critical phenomenon for droplet concentration and should be considered in room design and placement of air conditioners.
3. Since the movement of droplets in the air seems chaotic due to turbulent airflow and each indoor environment has its variables, it is recommended to make safer indoor environment layout plans with CFD simulations instead of creating a safe distance.
4. The number of droplets has decreased over time in every situation due to either the droplets attaching to the surface or evaporating, but the rate of decrease in droplet count has varied in each situation. The structure of the airflow, which changes depending on the blowing speed, has directly affected the duration of droplets in the air.
5. While large-diameter droplets adhere quickly to the surface, small-diameter droplets can still remain in the air after 20 s, and the reduction in droplet number decreases after 10 s in all cases.
6. The air jet of the air conditioner directed most of the droplets to the right wall of the room. As a result, the droplets were kept away from the airflow in the room. This result is significant in terms of determining the risky areas in the room and showing that the SARS-CoV-2 infection risk can be reduced with room design and ventilation.
7. In all cases examined, it was observed that droplet spread was intense on the stretcher and the mannequin representing the doctor. The results indicate that the patient and doctor’s positions in the room can be set to reduce the infection risk. This finding showed the importance of room design and the use of additional protective equipment.

Healthcare workers are at the forefront of struggling with the outbreak. Safety arrangements that are significantly important for human health can be made in workplaces, such as hospital examination rooms, by investigating infectious droplet dispersion. The obtained findings can be used to revise hospital examination rooms for pandemic conditions or design appropriate ventilation systems in post-pandemic restructuring.
